# Evaluation of Learning Outcomes and Influencing Factors of Self-Directed Online Vaccination Education

**DOI:** 10.3390/vaccines14100872

**Published:** 2026-10-01

**Authors:** Wen-Yu Wang, Jia Shen, Lin Luan, Hai Jiang, Juan Xu, Ben-Feng Zheng, Yun-Yan Zhang, Zhuo-Yu Zhang, Na Liu

**Affiliations:** 1 Suzhou Center for Disease Control and Prevention, No. 16 Guangqian Road, Xiangcheng District, Suzhou 215000, China; wwy910625@outlook.com (W.-Y.W.); szluanlin@163.com (L.L.); xujuansuda@163.com (J.X.); zhengbenfeng36@126.com (B.-F.Z.); weiyizyy66@163.com (Y.-Y.Z.); sz_zhuoyu@163.com (Z.-Y.Z.); 2 Taicang City Center for Disease Control and Prevention, No. 20 Xingye South Road, Taicang, Suzhou 215400, China; shenjia04@126.com (J.S.); tccdccfk@126.com (H.J.)

**Keywords:** online parental vaccination education, vaccination, awareness rate of vaccine knowledge, effectiveness evaluation, before-and-after study

## Abstract

Vaccination is one of the most cost-effective public health interventions, yet parental knowledge gaps remain a barrier to timely childhood immunization. Digital health platforms offer new opportunities for scalable education. **Objective**: To evaluate the effectiveness of an online parental vaccination education program delivered via the “Suzhou Vaccination” WeChat official account in improving parents’ knowledge about vaccination, and to explore the application value of online education in parental health initiatives. **Methods**: A before-and-after study design was employed. Data were collected via questionnaires completed by parents before the implementation of the online program (late January 2023) and one year after implementation (early February 2024). The changes in the awareness rate of vaccine knowledge (scores) and influencing factors were analyzed and compared. **Results**: A total of 6029 pre-intervention and 3790 post-intervention valid questionnaires were included. The pass rate of knowledge awareness after the intervention (55.57%, 2106/3790) was statistically higher than that before the intervention (52.33%, 3155/6029) (*p* < 0.05). Among the 10 knowledge items, the difference in correct response rates for 5 items was statistically significant (*p* < 0.05) before and after the intervention. The correct response rates for three items significantly increased (50.29% vs. 52.93%; 63.23% vs. 67.65%; 83.73% vs. 85.36%), while the rates for two items significantly decreased (92.50% vs. 90.71%; 53.11% vs. 49.68%); all these changes were statistically significant (*p* < 0.05). Multivariate logistic regression identified six independent factors significantly associated with higher post-intervention knowledge scores (all *p* < 0.05): younger child’s age (0–2 years), parent age ≥ 26 years, university education or above, employment as teachers/medical professionals/civil servants, higher household income (≥¥150,000), and being the child’s mother. **Conclusions**: The online parental education platform was associated with a statistically significant but modest improvement in parents’ vaccination knowledge, and the child’s age, the respondent’s age, occupation, education level, annual household income, and relationship to the child influenced the post-intervention knowledge scores.

## 1. Introduction

Vaccination is widely regarded as one of the greatest public health strategies and most cost-effective preventive services of the 20th century, as well as the most efficient and economical means for preventing and controlling infectious diseases [1]. Parental vaccination education is an important approach that mobilizes societal participation and utilizes various methods for health education, aiming to improve guardians’ knowledge about vaccination [2].

However, with evolving times, traditional community-based, in-person parental education sessions face significant challenges due to constraints in staffing, time, venue and other condition [3]. While these sessions offer face-to-face interaction, a segment of parents is unable to attend due to various constraints, consequently missing opportunities to acquire essential vaccination knowledge and creating gaps in the coverage of vaccination education. Online learning offers greater flexibility in both time and space [4]. Furthermore, in the context of prevailing infectious diseases, gathering in person can potentially facilitate the transmission of respiratory illnesses [5].

As an effective instant communication tool, WeChat has been extensively applied in the medical field [6]. With continuous digital advancement, the integration of digital tools into vaccination services is becoming a prevailing trend [5,7], and the digital empowerment of immunization programs has become an emerging trend, highlighting the advantages of online parental education [8]. Utilising multi-media information platforms to provide vaccination knowledge through diverse, targeted channels has emerged as a novel approach [9]. In recent years, online parental education, as a new form of health communication, has demonstrated positive outreach outcomes in cities like Shanghai and Nanchang [3,10]. Internationally, randomized controlled trials in Italy and other countries have shown that smartphone apps, websites, and AI-powered chatbots are effective in increasing parental vaccine knowledge, confidence, and uptake [11]. In regions with significant migrant populations or remote areas, the need to intensify vaccination-related health education through information technology and the internet is increasingly pressing [12]. Online parental education platforms can serve as an effective means to address this need.

Several factors are known to influence the effectiveness of parental education programs, including participants’ education level, geographic region, and relationship to the child (e.g., mother, father, or other guardian) [13]. Understanding these factors is essential for optimizing the design and implementation of online parental education and for interpreting the results of effectiveness evaluations. In addition, WeChat-based parental vaccination education is user-friendly, reaches a broad audience, and disseminates information rapidly, allowing vaccine recipients and their guardians to utilize fragmented time for learning relevant courses anytime and anywhere [3].

Although online parental education platforms have been implemented in several Chinese cities, limited evidence exists regarding their real-world effectiveness in improving vaccination knowledge among parents of young children. Furthermore, few studies have conducted item-level analyses to identify specific knowledge domains that are most—and least—effectively addressed by such interventions.

On 1 February 2023, an online parental vaccination education platform was officially launched via the “Suzhou Vaccination” WeChat official account, serving users in Suzhou City. This study analyzes changes in vaccination knowledge awareness and the effectiveness of the education platform before and after its implementation, explores the application value of online education in parental vaccination initiatives, and provides new insights for future formats of parental education.

## 2. Materials and Methods

### 2.1. Study Design

This study employed a before-and-after study design, with surveys conducted at two time points: before the implementation of the online parental education platform (late January 2023) and one year after its implementation (early February 2024).

As this study was designed as a pragmatic evaluation of a real-world online education platform, no a priori sample size calculation was performed. The sample size was determined by the number of parents who voluntarily accessed and completed the survey during the study period.

The questionnaire was developed specifically for this study based on the literature and the characteristics of the target population and was reviewed by expert colleagues [14,15,16]. It was not formally validated in a pilot study.

Due to the anonymous and voluntary nature of the online survey distributed via the official account, and because the ‘Questionnaire Star’ platform does not record the number of unique users who accessed the survey link, a precise response rate could not be calculated.

### 2.2. Study Population and Selection Criteria

Inclusion criteria were: users with an IP address located within Jiangsu Province during the survey period who were guardians of children aged 0–6 years. Exclusion criteria were: users without a child aged 0–6 years; those with an IP address outside Jiangsu Province during the survey period; and those with repeatedly duplicated IP addresses.

### 2.3. Data Collection

#### 2.3.1. General Information Questionnaire

A general information questionnaire collected basic demographic data, including the age of the child in the household, the respondent’s relationship to the child (parental role), the respondent’s age, occupation, education level, and annual household income.

#### 2.3.2. Vaccination Knowledge Questionnaire

This questionnaire consisted of 10 items, which covered the curriculum of the online parental education platform, categorized into three domains (Supplementary File S1):

Items Q1, Q3, Q8, and Q10 related to precautions before and after vaccination.

Items Q2, Q4, and Q7 related to the Bacille Calmette-Guérin (BCG) vaccine.

Items Q5, Q6, and Q9 related to the vaccine use recommendations.

Each item was scored 10 points for a correct answer and 0 points for an incorrect or unanswered question. A score of 80 points or higher was defined as “Adequate Knowledge of Vaccination” [15,16].

### 2.4. Survey Methodology

The survey was administered via the “Suzhou Vaccination” WeChat official account. A hyperlink or a QR code redirecting to the questionnaire (hosted on “Questionnaire Star,” a popular Chinese online survey platform) were embedded in push notification articles. Clicking the hyperlink or scanning the QR code with a smartphone directed users to the online questionnaire. Both methods opened the same questionnaire in a web browser, and participants could complete it on their mobile devices. The estimated completion time was 5 min, and each WeChat account was restricted to a single submission. To enhance data accuracy, logical checks and skip patterns were incorporated into the questionnaire for quality control. Upon survey completion, data were exported from the “Questionnaire Star” into Excel spreadsheets for verification and organization.

The study was reviewed and approved by the Ethics Committee of Suzhou Center for Disease Control and Prevention (NO. SZJKMG2023-005, 10 January 2023). Informed consent was obtained electronically from all participants before they proceeded to the questionnaire.

### 2.5. Statistical Analysis

Data were compiled using Microsoft Excel and analyzed with IBM SPSS Statistics for Windows, version 23.0. Categorical data, including awareness rate of vaccine knowledge, are presented as percentages (%). Inter-group comparisons were performed using the Chi-square test. Multivariate logistic regression was used to identify factors associated with post-intervention knowledge scores. For each categorical variable, the reference group was chosen as the category expected to have the lowest odds of adequate knowledge based on prior literature and clinical reasoning (e.g., younger age group, lower education level, lower income level). A *p*-value of less than 0.05 was considered statistically significant.

## 3. Results

According to the inclusion criteria, a total of 10,910 questionnaires were initially collected, comprising 6700 before and 4210 after the implementation of the online parental vaccination education program. After excluding 1091 questionnaires based on the exclusion criteria, 9819 questionnaires were ultimately included in the analysis, with 6029 from the pre-intervention group and 3790 from the post-intervention group.

### 3.1. Baseline Characteristics of the Study Participants

The majority of the children in the households were aged 0–2 years, accounting for 44.30% of the sample. Most respondents were the children’s mothers (61.58%). The respondents were predominantly aged between 26 and 35 years (70.61%). Their occupations were primarily in commerce and services (56.52%), and the highest education level for most was university or above (73.59%). The annual household income was mostly concentrated between 50,000 and 150,000 RMB. No significant differences were observed in the children’s age, the respondent’s relationship to the child, respondent’s age, occupation, education level, or annual household income between the pre- and post-intervention groups (Table 1).

### 3.2. Analysis of the Online Parental Education Platform’s Effectiveness

The total response time for survey respondents, both before and after the intervention, ranged between 13 and 300 s. Normality tests indicated that the distribution of response times was normal for both groups. The mean response time (±standard deviation) was 164.61 ± 71.44 s in the pre-intervention group and 163.58 ± 71.15 s in the post-intervention group. No significant difference was found in the time taken to complete the questionnaire.

The proportion of participants with adequate knowledge was 52.33% (3155/6029) in the pre-intervention group, which increased significantly to 55.57% (2106/3790) in the post-intervention group (χ^2^ = 9.803, *p* < 0.05). See Figure 1.

### 3.3. Analysis of Responses to Single Question Before and After the Intervention

A statistical analysis was performed on the answer choices for each question. The results indicated that for questions Q2, Q3, Q4, Q7, and Q9, both the correct response rate and the distribution of all answer choices were significantly different post-intervention (*p* < 0.05). For questions Q5 and Q8, no significant change was found in the correct rate, but a significant difference was observed in the distribution of the answer selections (*p* < 0.05). A detailed comparison is provided in Table 2.

Statistical analysis revealed significant differences in the correct response rates for five out of the ten items (*p* < 0.05): Q2, Q3, Q4, Q7, and Q9. The correct rates for Q2, Q4, and Q7, which pertain to knowledge about the BCG vaccine, showed significant improvement (50.29% vs. 52.93%; 63.23% vs. 67.65%; 83.73% vs. 85.36%, respectively). Conversely, the correct rates for Q3 and Q9, which relate to vaccination precautions and vaccine usage recommendations, showed a significant decline (92.50% vs. 90.71%; 53.11% vs. 49.68%, respectively). See Figure 2.

### 3.4. Analysis of Factors Influencing Post-Intervention Knowledge Scores

In this analysis, the dependent variable was the post-intervention knowledge score, dichotomized as 1 (<80 points) and 2 (≥80 points). Based on the variable assignments in Table 1, univariate logistic regression identified six factors with a statistically significant association with the knowledge score (*p* < 0.05): the child’s age, the respondent’s age, occupation, education level, annual household income, and relationship to the child. These significant factors were subsequently included in a multivariate logistic regression model, which confirmed that all six remained independent and statistically significant predictors of the knowledge score (*p* < 0.05). The higher post-intervention knowledge scores are significantly associated with having a child aged 0–2 years, parent age ≥ 26 years, university education or above, employment as a teacher, medical professional, or civil servant, higher household income (≥¥150,000), and being the child’s mother. The results of the multivariate analysis are presented in Table 3.

## 4. Discussion

Improving vaccination knowledge among parents plays a crucial role in enhancing their engagement with childhood immunisation, thereby positively promoting timely vaccination coverage [17,18]. In recent years, as societal emphasis on childhood vaccination has grown, so has parents’ demand for related knowledge [19].

This study evaluated the real-world effectiveness of a WeChat-based online parental education platform in improving vaccination knowledge among parents of children aged 0–6 years in Suzhou, China.

The study by Chen Peng et al. focused on general vaccination knowledge awareness but did not categorize the specific domains of the survey items [13]. This study, however, employed a carefully designed questionnaire with items categorised into domains such as the BCG vaccine, vaccination precautions, and vaccine usage recommendations, thereby conveying comprehensive knowledge from multiple perspectives.

**(1) BCG-Related Knowledge**

The Bacille Calmette-Guérin (BCG) vaccine, due to its unique biological characteristics, complex administration schedule, and significant public health importance, remains one of the most focal vaccines [20]. Items Q2, Q4, and Q7 pertained to BCG knowledge, covered in the online courses “Which Two Vaccines Does a Baby Need at Birth?” and “Vaccination is as Important as Breastfeeding.”

Post-intervention, for Q2 (disease prevented by BCG), the proportion of respondents incorrectly selecting “Poliomyelitis” decreased significantly, while the proportion correctly selecting “Miliary Tuberculosis and Tuberculous Meningitis” increased markedly.

For Q4 (incorrect statement about BCG), the proportion of respondents selecting the distractor “If the abscess/ulcer diameter exceeds 1 cm or persists >3 months, seek care at a BCG reaction clinic” decreased significantly. Conversely, the proportion correctly identifying the true statement regarding the management of a typical local pustule increased. These findings indicate a marked improvement in participants’ understanding of BCG following the online education.

For Q7 (first vaccines after birth), the proportion of respondents incorrectly selecting “Polio and Hepatitis B vaccine” or “Pneumococcal and BCG vaccine” decreased post-intervention, while the proportion correctly selecting “BCG and Hepatitis B vaccine” increased.

These findings are consistent with other studies [10,21], who reported that comprehensive health education plays a significant role in disseminating more in-depth and specialized knowledge among the general population.

**(2) Knowledge on Vaccination Certificate, Observation Period, and Contraindications**

Items Q1 and Q8 concerned the vaccination certificate, Q3 the post-vaccination observation period, and Q10 temporary contraindications for vaccination; all were addressed in the course “What to Pay Attention to Before and After Vaccination.”

Post-intervention, the correct response rates for Q1 and Q8 remained high (>80%) and unchanged. This finding differs from that of Liao et al. [10], who reported a significant increase in correct response rates after an online educational intervention. A plausible explanation is that participants in our study already had a high baseline level of knowledge regarding vaccination certificates, resulting in a ceiling effect and leaving limited room for further improvement. However, the correct rate for Q3 (30-min observation) decreased significantly, with an increase in respondents selecting the incorrect “15 min.” This suggests that the critical information regarding the recommended observation period was not sufficiently emphasised in the course content and requires strengthening in future iterations.

For Q10 (multiple-choice on contraindications), no significant improvement was observed in the overall correct rate, and the selection patterns for individual options showed no significant shift. This finding is consistent with a study by Jiang et al. [21], which also reported only limited improvement in vaccination contraindication knowledge even after an educational intervention. This indicates that education regarding temporary contraindications needs enhancement in the curriculum, and more innovative communication methods should be explored.

**(3) Knowledge on Vaccine Types, Hepatitis B, and Combination Vaccines**

Items Q5 concerned vaccine categories (EPI vs. non-EPI), Q6 the Hepatitis B vaccine schedule, and Q9 combination vaccines; these were covered in the courses “Which Two Vaccines Does a Baby Need at Birth?” and “Combination Vaccines.”

The correct response rate for Q6 (Hepatitis B doses) was high both before and after the intervention, with no significant change, likely due to a ceiling effect. In contrast, the correct rate for Q5 (correct statement about vaccine categories) was low at both time points and showed no significant improvement, which is inconsistent with previous studies where targeted education improved parental knowledge of vaccine categories [10,21]. This suggests that the current course content should be further strengthened with more targeted teaching on vaccine categories, particularly the value of non-EPI vaccines.

For Q9 (correct statement about combination vaccines), although the overall correct rate decreased post-intervention, the distribution of answer choices shifted significantly. The proportion of respondents selecting the distractor “They are single vaccines containing a simple mixture of components…” increased, indicating participants grasped the concepts of “multiple components” and “single vaccine” from the course but overlooked the inaccuracy of “simple mixture.” This suggests that future courses should more explicitly clarify what constitutes a combination vaccine and emphasize the importance of precise definitions. Although this shift did not translate into a higher correct rate, it indicates that the online education did influence participants’ understanding of combination vaccines.

This study found that the proportion of participants with adequate vaccination knowledge significantly increased one year after the implementation of the online parental education platform, a result consistent with findings from other regions [3,6].

Post-intervention knowledge scores were influenced by several factors, including the child’s age, the respondent’s age, occupation, education level, annual household income, and relationship to the child. Higher scores were associated with having a child aged 0–2 years, being aged 26–35 years, holding a university degree or higher, working as a teacher, medical professional, or civil servant, having an annual household income ≥¥150,000, and being the child’s mother, which aligns with the findings of reference [14]. This suggests that future vaccination education initiatives should pay particular attention to reaching populations with lower education levels and those employed in industrial or agricultural sectors [22].

The Suzhou online parental education platform was officially launched on 1 February 2023, prior to which parental education was conducted solely via in-person sessions. In 2022, the number of parents reached by these sessions accounted for 67.53% of the birth cohort that year. In contrast, following the platform’s introduction in 2023, the participation rate surged to 93.50% of the birth cohort. This substantial increase demonstrates that the online model is more effective at engaging parents. It facilitates easier access to knowledge for parents while simultaneously reducing the workload for vaccination clinic staff, allowing them to dedicate more energy to other essential immunization tasks. This, in turn, has a positive impact on improving the overall vaccination service environment [23].

This study possesses several innovative aspects. It employed a pre-post design to analyze changes in participants’ scores and their influencing factors. Furthermore, to gain deeper insights into the acquisition of specific vaccination knowledge points, we compared the selection patterns for all options of individual items before and after the intervention. This detailed analysis of knowledge shifts provides a solid evidence base for further refining the online parental education platform.

However, this study has certain limitations. Firstly, the study didn’t perform a priori sample size calculation, as the study was designed as a pragmatic evaluation of an ongoing public health intervention. The final sample size (n = 9819) was sufficiently large to provide robust statistical power for all analyses. Therefore, we do not consider the absence of an a priori sample size calculation to be a limitation of this study. Secondly, the participants were users following the “Suzhou Vaccination” WeChat official account, which may limit the generalizability of the findings. Additionally, administering the questionnaire via “Questionnaire Star” might have introduced interference from automated ‘bot’ accounts, potentially leading to a higher proportion of invalid responses. Thirdly, the questionnaire used in this study was not formally validated in a pilot study, which may limit the measurement validity of our findings. However, the items were derived from established vaccination knowledge frameworks and reviewed by expert colleagues at the Suzhou CDC. Future studies should consider formal validation of the instrument to further strengthen the evidence base.

Despite these limitations, this study offers a new perspective for conducting parental education. It suggests that in routine practice, data from online learning could be synchronized with community health centers, and parents with low knowledge scores could be offered supplementary offline learning sessions [24]. Moreover, beyond using WeChat platforms, developing dedicated mobile applications (Apps) for vaccination could also effectively improve parental knowledge and timely vaccination rates [8,25].

## 5. Conclusions

The online parental education platform represents a novel approach aligned with contemporary development. It can provide parents with quicke access to essential knowledge and significantly improves their vaccination literacy, serving as a crucial supplement to in-person sessions. Given the large sample size, this finding should be interpreted with attention to its practical significance. Even small improvements in knowledge at the population level may contribute to better vaccination coverage and timeliness. A combined online and offline model is more effective in enhancing parental awareness and engagement, holding profound significance for the effective control of vaccine-preventable diseases.

## Figures and Tables

**Figure 1:**
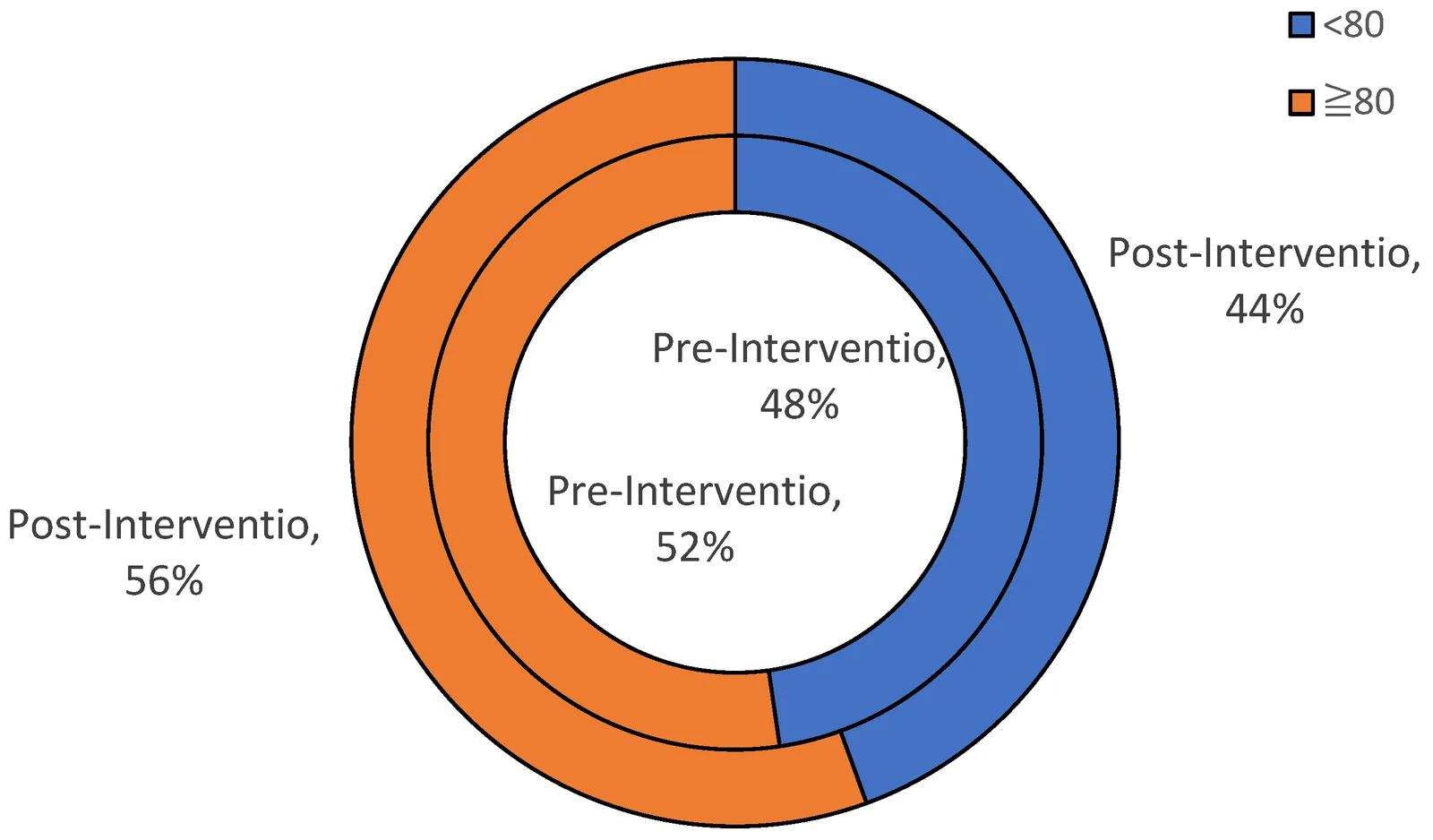
Vaccination Knowledge Status of Survey Participants Before and After the Online Parental Vaccination Education Platform Intervention.

**Figure 2:**
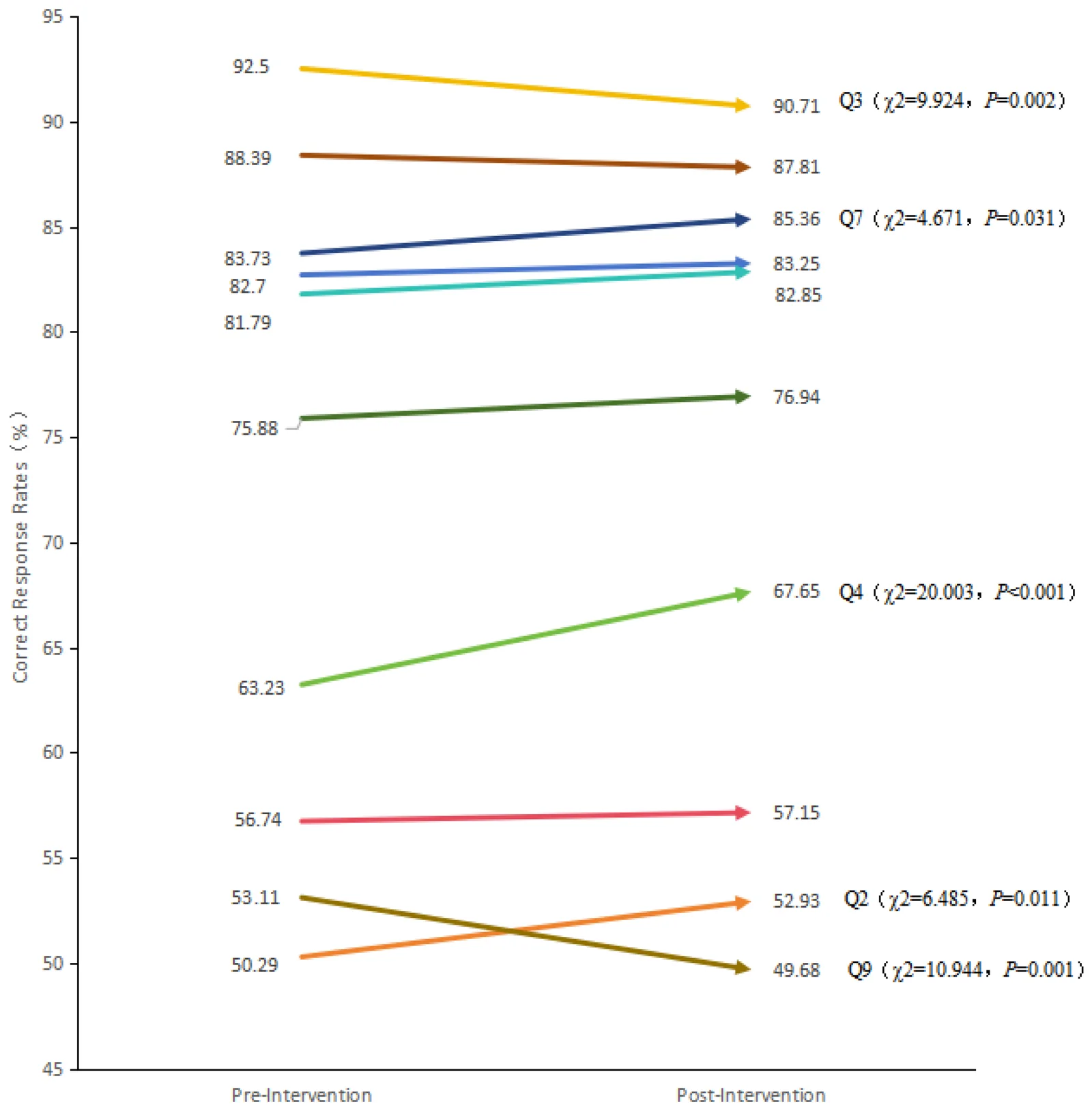
Comparison of Correct Response Rates for Each Question Before and After the Online Parental Education Platform Intervention. Note(s): 1. Unanswered questions were scored as incorrect. 2. Only items with statistically significant differences (*p* < 0.05) are indicated in the figure. 3. The arrows indicate the direction of change from pre- to post-intervention. Colors are used to differentiate individual questions.

**Table 1:** Baseline Characteristics of Participants Before and After the Online Parental Vaccination Education Platform Intervention.

Variable	Assignment *	Pre-Intervention		Post-Intervention		χ^2^	*p*-Value
		*N*	Proportion (%)	*N*	Proportion (%)		
Child’s age (years)							
≤2	1	2693	44.67	1679	44.30	0.127	0.938
3–5	2	2357	39.09	1491	39.34		
≥6	3	979	16.24	620	16.36		
Relationship to child							
Father	1	2086	34.60	1367	36.07	2.205	0.332
Mother	2	3799	63.01	2334	61.58		
Other guardian	3	144	2.39	89	2.35		
Respondent’s age (years)							
≤25	1	394	6.54	240	6.33	0.479	0.787
26–35	2	4277	70.94	2676	70.61		
≥36	3	1358	22.52	874	23.06		
Occupation							
Teacher, Medical staff, Civil servant	1	1131	18.76	717	18.92	2.225	0.329
Worker, Migrant worker, Farmer	2	1408	23.35	931	24.56		
Commerce, Services, Others	3	3490	57.89	2142	56.52		
Education Level							
Primary school or below	1	230	3.81	163	4.30	1.609	0.447
Junior/High school or Secondary specialist school	2	1318	21.86	838	22.11		
University or above	3	4481	74.32	2789	73.59		
Annual Household Income (¥10,000)							
<5	1	428	7.10	294	7.76	5.448	0.066
5–15	2	3291	54.59	1980	52.24		
≥15	3	2310	38.31	1516	40.00		

* Variable assignments were used for the subsequent logistic regression analysis.

**Table 2:** Distribution of Answers for Each Question Before and After the Intervention.

Item & Options	Pre-Intervention		Post-Intervention		χ^2^	*p*-Value
	*N* *^1^	%	*N* *^1^	%		
Q1 How soon after a child’s birth should the guardian go to the local vaccination clinic to obtain the vaccination certificate?						
A. Within 1 month *^2^	4986	82.74	3155	83.29		
B. Within 2 months	483	8.02	316	8.34	2.388	0.303
C. Within 3 months	557	9.24	317	8.37		
Q2 Which disease does the BCG vaccine prevent?						
A. Poliomyelitis	2548	42.33	1480	39.12		
B. Miliary Tuberculosis and Tuberculous Meningitis *^2^	3032	50.37	2006	53.03	9.967	0.007
C. Pneumonia	439	7.29	297	7.85		
Q3 How long should one stay for observation after vaccination before leaving the facility?						
A. 10 min	197	3.27	111	2.94	20.888	0.000
B. 15 min	229	3.80	207	5.48		
C. 30 min *^2^	5577	92.60	3438	90.95		
D. Leave once the injection site stops bleeding	20	0.33	24	0.63		
Q4 Regarding BCG vaccination, which statement is INCORRECT?						
A. If the baby has severe conditions at birth, vaccination should be postponed until stable/cured.	934	15.55	612	16.23	40.768	0.000
B. A small local pustule (<0.5 cm) may appear ~2 weeks post-vaccination; apply hot compress, bandage, or disinfect with iodine/alcohol. *^2^	3812	63.48	2564	67.99		
C. If the abscess/ulcer diameter exceeds 1 cm or persists > 3 months, seek care at a BCG reaction clinic.	1259	20.97	595	15.78		
Q5 Regarding EPI (Expanded Program on Immunization) and non-EPI vaccines, which statement is CORRECT?						
A. Non-EPI vaccines are not needed.	519	8.67	374	9.94	21.058	0.000
B. Non-EPI vaccines effectively complement EPI vaccines, providing broader protection. *^2^	4931	82.33	3140	83.47		
C. EPI vaccines are self-paid.	539	9.00	248	6.59		
Q6 How many doses are typically required for the complete Hepatitis B vaccine series?						
A. 1 dose	325	5.44	217	5.77	1.225	0.747
B. 2 doses	1979	33.14	1232	32.78		
C. 3 doses *^2^	3421	57.28	2166	57.64		
D. 4 doses	247	4.14	143	3.81		
Q7 Which vaccine(s) is/are administered first after birth?						
A. BCG and Hepatitis B vaccine *^2^	5048	84.67	3235	86.17	9.408	0.009
B. Polio and Hepatitis B vaccine	636	10.67	391	10.42		
C. Pneumococcal and BCG vaccine	278	4.66	128	3.41		
Q8 Regarding the vaccination certificate, which statement is INCORRECT?						
A. It is an important document for school entry, nursery admission, and travelling abroad.	342	5.75	206	5.50	6.351	0.042
B. Only ID documents are needed for vaccination; the certificate is not required. *^2^	5329	89.67	3328	88.79		
C. It is a record of the child’s immunisations and should be kept safely.	272	4.58	214	5.71		
Q9 Regarding combination vaccines, which statement is CORRECT?						
A. They refer to administering multiple separate vaccines simultaneously.	774	13.12	528	14.17	12.940	0.002
B. They are single vaccines containing mixed components to prevent multiple diseases.	1923	32.60	1316	35.31		
C. Some have age limits and must be administered within specified ages. *^2^	3202	54.28	1883	50.52		
Q10 In which of the following situations should vaccination be postponed? (Multiple choice)						
A. Suffering from an acute illness *^2^	4938	81.90	3160	83.38	3.494	0.062
B. Acute exacerbation of a chronic disease *^2^	5067	84.04	3230	85.22	2.476	0.122
C. During a fever (axillary temperature ≥ 37.1 °C) *^2^	5277	87.53	3309	87.31	0.101	0.751

*^1^ n represents the number of respondents who selected each option. *^2^ Denotes the correct answer.

**Table 3:** Univariate and Multivariate Logistic Regression Analysis of Factors Associated with High Post-Intervention Scores following the Online Parental Education Platform.

Factor	OR (95%CI) *	
	Logistic (Simple)	Logistics (Multiple)
Child’s Age (years)		
≤2	1	1
3–5	0.462 (0.400, 0.534)	0.479 (0.410, 0.560)
≥6	0.342 (0.283, 0.414)	0.355 (0.289, 0.437)
Respondent’s Age (years)		
≤25	1	1
26–35	2.784 (2.187, 3.545)	1.834 (1.389, 2.422)
≥36	1.946 (1.495, 2.534)	1.834 (1.352, 2.487)
Occupation		
Teacher, Medical staff, Civil servant	1	1
Worker, Migrant worker, Farmer/Fisher	0.436 (0.357, 0.533)	0.753 (0.600, 0.945)
Commerce, Services, Others	0.749 (0.628, 0.892)	0.845 (0.697, 1.025)
Education Level		
Primary school or below	1	1
Junior/High school or Secondary specialist school	6.809 (3.798, 12.207)	4.214 (2.315, 7.672)
University or above	20.419 (11.525, 36.174)	9.642 (5.323, 17.465)
Annual Household Income (¥10,000)		
<5	1	1
5–15	1.143 (0.893, 1.462)	0.817 (0.619, 1.079)
≥15	2.613 (2.027, 3.369)	1.386 (1.031, 1.863)
Relationship to Child		
Father	1	1
Mother	2.103 (1.836, 2.409)	1.935 (1.664, 2.250)
Other guardian	0.850 (0.550, 1.316)	1.101 (0.680, 1.781)

* Note: CI = Confidence Interval. OR = Odds Ratio.

## Data Availability

The raw data supporting the conclusions of this article will be made available by the authors on request, due to privacy and ethical restrictions.

## Supplementary Materials

The following supporting information can be downloaded at https://www.mdpi.com/article/10.3390/vaccines14100872/s1, File S1: Questionnaire.
