# Supplementary material for: Evaluation of Learning Outcomes and Influencing Factors of Self-Directed Online Vaccination Education
Source: Vaccines (Basel). 2026 Oct 1;14(10):872. doi: 10.3390/vaccines14100872 (PMC13709408; doi:10.3390/vaccines14100872)
Supplement: Supplementary file 1 [file vaccines-14-00872-s001.zip › vaccines-4544315-supplementary.pdf]

File S1: Questionnaire.

1. General Information

Q1 **What is the age of your child? (years)(If you have multiple children, please provide the age of the younger one.)** ________

Q2 **You are the child's:**

A.Father

B.Mother

C.Other guardian

Q3 **What is the age of the respondent? (years)**

A.≦25

B.26-35

C.≥36

Q4 **What is your occupation? (If retired, please indicate your former occupation.)**

A.Teacher, Medical staff, Civil servant

B.Worker, Migrant worker, Farmer

C.Commerce, Services, Others

Q5 **What is your highest level of education?**

A.Primary school or below

B.Junior/High school or Secondary specialist school

C.University or above

Q6 **What is your annual household income level (in CNY 10,000)?**

A.<5

B.5-15

C.≥15

2.Vaccination Knowledge

Q1 **How soon after a child's birth should the guardian go to the local vaccination clinic to obtain the vaccination certificate?**

A.Within 1 month*[2]

B.Within 2 months

C.Within 3 months

Q2 **Which disease does the BCG vaccine prevent?**

A.Poliomyelitis

B.Miliary Tuberculosis and Tuberculous Meningitis*

C.Pneumonia

Q3 **How long should one stay for observation after vaccination before leaving the facility?**

A.10 minutes
B.15 minutes
C.30 minutes*
D.Leave once the injection site stops bleeding

Q4 **Regarding BCG vaccination, which statement is INCORRECT?**
A. If the baby has severe conditions at birth, vaccination should be postponed until stable/cured.
B.A small local pustule (<0.5cm) may appear ~2 weeks post-vaccination; apply hot compress, bandage, or disinfect with iodine/alcohol.*
C.If the abscess/ulcer diameter exceeds 1cm or persists >3 months, seek care at a BCG reaction clinic.

Q5 **Regarding EPI (Expanded Program on Immunization) and non-EPI vaccines, which statement is CORRECT?**
A.Non-EPI vaccines are not needed.
B.Non-EPI vaccines effectively complement EPI vaccines, providing broader protection.*
C.EPI vaccines are self-paid.

Q6 **How many doses are typically required for the complete Hepatitis B vaccine series?**
A.1 dose
B.2 doses
C.3 doses*
D.4 doses

Q7 **Which vaccine(s) is/are administered first after birth?**
A.BCG and Hepatitis B vaccine*
B.Polio and Hepatitis B vaccine
C.Pneumococcal and BCG vaccine

Q8 **Regarding the vaccination certificate, which statement is INCORRECT?**
A.It is an important document for school entry, nursery admission, and travelling abroad.
B.Only ID documents are needed for vaccination; the certificate is not required.*
C.It is a record of the child's immunisations and should be kept safely.

Q9 **Regarding combination vaccines, which statement is CORRECT?**
A.They refer to administering multiple separate vaccines simultaneously.
B.They are single vaccines containing mixed components to prevent multiple diseases.
C.Some have age limits and must be administered within specified ages.*

Q10 **In which of the following situations should vaccination be postponed? (Multiple choice)**
A.Suffering from an acute illness*
B.Acute exacerbation of a chronic disease*

C.During a fever (axillary temperature ≥37.1°C)*

*[2]Denotes the correct answer.
